# Fatores de Risco Associados à Pré-Eclâmpsia e Seu Impacto na Morbidade e Mortalidade Materna e Neonatal na República Dominicana

**DOI:** 10.36660/abc.20260474

**Published:** 2026-08-03

**Authors:** Patricia Severino, Fulgencio Severino, Rosa Cueto

**Affiliations:** 1 Hospital Dr Salvador B Gautier Santo Domingo República Dominicana Hospital Dr Salvador B Gautier – Cardiología, Santo Domingo – República Dominicana; 2 Sociedad Dominicana de Cardiología Santo Domingo República Dominicana Sociedad Dominicana de Cardiología, Santo Domingo – República Dominicana

**Keywords:** Pré-Eclâmpsia, Hipertensão Induzida pela Gravidez, Mortalidade Materna, Recém-Nascido Prematuro, Recém-Nascido de Baixo Peso, República Dominicana

## Abstract

**Fundamento:**

Os distúrbios hipertensivos da gestação complicam 5%-10% das gestações e são responsáveis por 23%-38% das mortes maternas na República Dominicana.

**Objetivo:**

Identificar os fatores associados ao desenvolvimento da pré-eclâmpsia e avaliar sua relação com a morbidade e mortalidade materna e perinatal.

**Métodos:**

Este estudo multicêntrico, prospectivo, observacional, transversal e analítico incluiu 469 parturientes de 17 hospitais da República Dominicana durante um período de 30 dias. A significância estatística foi estabelecida em p < 0,05.

**Resultados:**

A pré-eclâmpsia esteve significativamente associada à hipertensão crônica (44,15% vs 16,87%; *odds ratio* [OR], 3,91; intervalo de confiança de 95% [IC95%], 2,51-6,09), à pré-eclâmpsia prévia (44,64% vs 18,60%; OR, 3,53; IC95%, 2,24-5,56) e ao histórico familiar de pré-eclâmpsia (42,0% vs 19,38%; OR, 3,01; IC95%, 1,96-4,62). A pré-eclâmpsia também esteve associada à prematuridade (54,6% vs 33,4%; OR, 2,40; IC95%, 1,62-3,55), ao baixo peso ao nascer (37,6% vs 18,5%; OR, 2,65; IC95%, 1,73-4,07) e ao descolamento prematuro de placenta (5,0% vs 2,4%; OR, 2,14; IC95%, 0,81-5,62). A mortalidade materna e fetal não esteve significativamente associada à pré-eclâmpsia. Na análise de regressão logística multivariável, hipertensão crônica (OR ajustada [AOR], 3,64; IC95%, 2,25-5,88), histórico familiar de pré-eclâmpsia (AOR, 2,86; IC95%, 1,85-4,42) e obesidade pré-gestacional (AOR, 2,26; IC95%, 1,52-3,36) emergiram como preditores independentes de pré-eclâmpsia (todos p < 0,05).

**Conclusão:**

A pré-eclâmpsia está associada a um risco aumentado de desfechos maternos e neonatais adversos, particularmente prematuridade e baixo peso ao nascer.

## Graphical abstract

**Figura Central:**
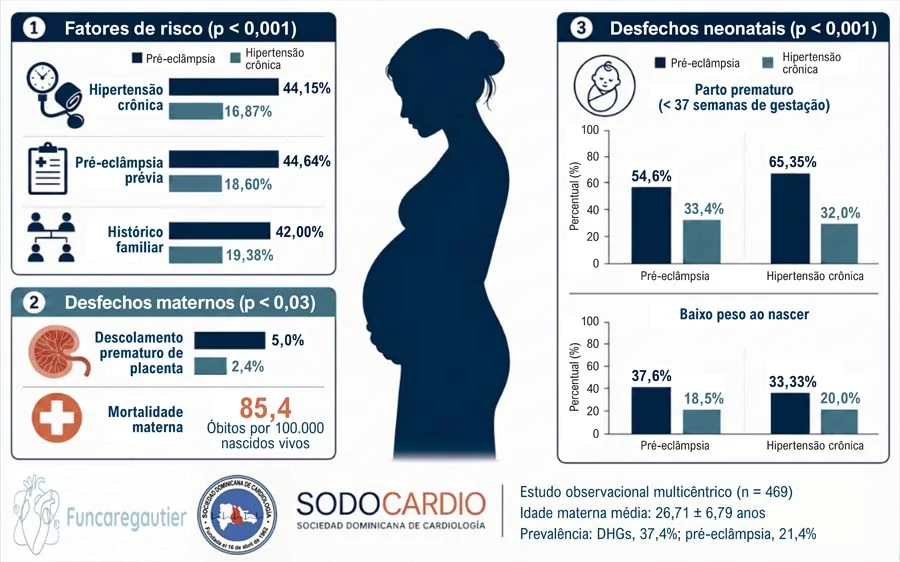
: Fatores de Risco Associados à Pré-Eclâmpsia e Seu Impacto na Morbidade e Mortalidade Materna e Neonatal na República Dominicana

DHGs: distúrbios hipertensivos da gestação.

## Introdução

Os distúrbios hipertensivos da gestação (DHGs) complicam aproximadamente 5% a 10% das gestações e representam um importante desafio de saúde pública, particularmente em países de baixa e média renda.^1-4^ Os DHGs estão associados a complicações obstétricas graves, incluindo parto prematuro, restrição do crescimento fetal, descolamento prematuro de placenta e mortalidade materna e fetal.^5,6^

Globalmente, os DHGs afetam aproximadamente 13%-15% das gestações e constituem um importante fator contribuinte para parto prematuro, restrição do crescimento fetal e sequelas cardiovasculares e metabólicas de longo prazo nas mulheres afetadas.^1,7^ Entretanto, seu impacto varia substancialmente entre os diferentes contextos. Em países de alta renda, como os Estados Unidos, os DHGs são responsáveis por aproximadamente 8%-9% das mortes maternas, enquanto na República Dominicana respondem por uma estimativa de 23%-38% das mortes maternas, de acordo com dados epidemiológicos do Serviço Nacional de Saúde (SNS) da República Dominicana.^2-4^ Essa disparidade reflete a influência substancial dos determinantes sociais da saúde, da vulnerabilidade socioeconômica e das iniquidades no acesso ao pré-natal. Apesar dessa elevada carga da doença, estudos multicêntricos recentes que caracterizem os fatores de risco associados aos DHGs e seu impacto nos desfechos maternos e neonatais permanecem escassos. Essa ausência de evidências robustas e específicas para o contexto limita a aplicabilidade das diretrizes clínicas internacionais ao sistema de saúde da República Dominicana e dificulta o desenvolvimento de estratégias eficazes de saúde pública para prevenção e manejo.

A identificação dos fatores clínicos e obstétricos associados à pré-eclâmpsia é essencial para otimizar a prevenção, a estratificação de risco e o manejo clínico oportuno.^3,5^ Estudos anteriores identificaram histórico de pré-eclâmpsia, hipertensão crônica, diabetes melito pré-gestacional, doença renal crônica, doenças autoimunes (particularmente lúpus eritematoso sistêmico e síndrome antifosfolípide) e gestação multifetal como os principais fatores de risco para o desenvolvimento de pré-eclâmpsia. A identificação precoce de mulheres com risco aumentado possibilita a implementação de intervenções preventivas, vigilância pré-natal mais rigorosa e tratamento oportuno, reduzindo, assim, a morbidade e mortalidade materna e neonatal.^6,8,9^

Evidências crescentes indicam que a pré-eclâmpsia não é apenas uma complicação obstétrica aguda, mas também um importante marcador de risco cardiovascular materno futuro, com mulheres afetadas apresentando maior risco de desenvolver hipertensão crônica, doença cardíaca isquêmica, insuficiência cardíaca e doença cerebrovascular posteriormente na vida.^10-12^ Da mesma forma, a pré-eclâmpsia está associada a desfechos neonatais adversos, incluindo parto prematuro, baixo peso ao nascer, admissão em unidade de terapia intensiva neonatal e mortalidade perinatal.^13-15^

Portanto, este estudo teve como objetivo identificar os fatores associados ao desenvolvimento da pré-eclâmpsia e avaliar a associação entre os DHGs e a morbidade e mortalidade materna e neonatal em uma rede hospitalar multicêntrica representativa da República Dominicana.

## Métodos

### Delineamento do estudo e população

Este estudo multicêntrico, observacional, analítico e transversal foi conduzido com coleta prospectiva de dados. A população do estudo foi composta por todas as gestantes admitidas nas unidades de trabalho de parto e parto de 17 hospitais pertencentes ao SNS, incluindo hospitais provinciais e centros nacionais de referência. Os dados foram coletados durante um período fixo de 30 dias, iniciado com a inclusão das pacientes em cada centro participante. Foi utilizada uma estratégia de amostragem por conveniência, sendo incluídas todas as pacientes elegíveis que atenderam aos critérios de inclusão durante o período do estudo.

### Critérios de elegibilidade

Foram elegíveis para inclusão as parturientes atendidas em um dos hospitais participantes que forneceram voluntariamente o termo de consentimento livre e esclarecido por escrito. Foram excluídas do estudo as pacientes que recusaram participar ou cujos prontuários continham dados essenciais ausentes ou informações insuficientes para verificar as principais variáveis do estudo.

### Definições e coleta de dados

A hipertensão foi definida como pressão arterial sistólica ≥ 140 mmHg e/ou pressão arterial diastólica ≥ 90 mmHg, documentada em duas medidas distintas obtidas com intervalo mínimo de 4 horas. A hipertensão crônica foi definida como hipertensão diagnosticada antes da gestação ou antes de 20 semanas de gestação. A pré-eclâmpsia foi definida de acordo com critérios diagnósticos estabelecidos como hipertensão de início recente após 20 semanas de gestação, acompanhada de proteinúria ou evidências de disfunção de órgãos-alvo maternos. A pré-eclâmpsia sobreposta foi definida como o desenvolvimento de proteinúria de início recente, piora súbita da pressão arterial previamente controlada ou novas evidências de disfunção de órgãos-alvo em mulheres com hipertensão crônica preexistente. A eclâmpsia foi definida como a ocorrência de crises convulsivas tônico-clônicas generalizadas em uma mulher com DHG que não pudessem ser explicadas por uma condição neurológica preexistente. O diabetes melito gestacional foi definido como glicemia plasmática de jejum ≥ 126 mg/dl em pelo menos duas medidas obtidas durante a segunda metade da gestação em mulheres sem diagnóstico prévio de diabetes melito. O baixo peso ao nascer foi definido como peso ao nascer < 2.500 g.

Os dados foram coletados por meio de formulários padronizados de relato de caso, utilizando entrevistas diretas com as participantes durante o período periparto e revisão abrangente dos prontuários médicos e exames complementares. A coleta de dados foi realizada por médicos treinados e estudantes de Medicina em fase final da graduação.

### Variáveis

As variáveis analisadas foram categorizadas em três domínios. As variáveis sociodemográficas incluíram idade materna, nacionalidade, escolaridade e situação ocupacional. As variáveis clínicas e obstétricas compreenderam hipertensão crônica, pré-eclâmpsia, eclâmpsia, diabetes melito preexistente, diabetes melito gestacional, obesidade, tabagismo, histórico familiar de pré-eclâmpsia e histórico prévio de pré-eclâmpsia. Os desfechos maternos e neonatais incluíram descolamento prematuro de placenta, parto prematuro (definido como parto com ≤ 37 semanas de gestação), baixo peso ao nascer, óbito fetal intrauterino e mortalidade materna.

### Análise estatística

Os dados foram validados e analisados utilizando o IBM SPSS Statistics for Windows, versão 27 (IBM Corp., Armonk, NY, EUA). As variáveis contínuas foram expressas como média ± desvio padrão após avaliação da normalidade pelo teste de Shapiro-Wilk. As variáveis categóricas foram resumidas em frequências e porcentagens.

Na análise bivariada, as associações entre variáveis categóricas foram avaliadas pelo teste do qui-quadrado de Pearson, enquanto as variáveis contínuas foram comparadas pelo teste *t* para amostras independentes. As variáveis que apresentaram significância estatística na análise bivariada ou que foram consideradas clinicamente relevantes foram incluídas em um modelo de regressão logística multivariável para identificar preditores independentes de pré-eclâmpsia. Foram calculadas as *odds ratios* ajustadas (AORs) e seus respectivos intervalos de confiança de 95% (IC95%). Todos os testes estatísticos foram bicaudais, sendo considerado estatisticamente significativo um valor de p < 0,05.

### Considerações éticas

O protocolo do estudo foi aprovado pelo Comitê de Ética em Pesquisa do Instituto Dominicano de Cardiologia e pelo SNS, órgão governamental responsável pela rede pública hospitalar da República Dominicana. Também foi obtida autorização administrativa dos diretores de todos os hospitais participantes.

Antes da inclusão, todas as participantes receberam informações detalhadas sobre os objetivos, procedimentos, potenciais benefícios e riscos do estudo, bem como sobre as medidas adotadas para garantir a confidencialidade e a proteção dos dados. Todas as informações coletadas foram utilizadas exclusivamente para fins de pesquisa, e nenhuma informação de identificação pessoal foi registrada. O termo de consentimento livre e esclarecido por escrito foi obtido de todas as participantes antes da inclusão no estudo.

## Resultados

### Características basais

Um total de 469 gestantes provenientes de diferentes regiões geográficas da República Dominicana foi incluído no estudo (Tabela 1). Os principais achados estão resumidos na Figura Central. A média de idade materna foi de 26,71 ± 6,79 anos. As características demográficas e clínicas basais são apresentadas na Tabela 2. A obesidade pré-gestacional foi o fator de risco basal mais prevalente (23,9%), seguida por hipertensão crônica (17,3%), histórico familiar de pré-eclâmpsia (12,0%) e histórico prévio de pré-eclâmpsia (9,0%). Diabetes melito gestacional e tabagismo ativo foram menos frequentes, acometendo 3,8% e 3,0% das participantes, respectivamente. No total, 37,4% das mulheres desenvolveram um DHG. A prevalência de pré-eclâmpsia foi de 21,4% (n = 100), enquanto a eclâmpsia ocorreu em 3,0% da população do estudo.

**Tabela 1:** – Hospitais participantes incluídos no estudo

Hospital	Província/localização
Hospital Materno Infantil San Lorenzo de Los Mina	Santo Domingo
Maternidade Nuestra Señora de la Altagracia	Distrito Nacional
Maternidade Evangelina Rodríguez	Distrito Nacional
Hospital Materno Infantil Reynaldo Almánzar	Santo Domingo
Hospital Dr. Marcelino Vélez Santana	Distrito Nacional
Hospital Juan Pablo Pina	San Cristóbal
Hospital Nuestra Señora de Regla	Peravia
Hospital Universitário Regional Jaime Mota	Barahona
Hospital Elio Fiallo	Pedernales
Hospital Luis Morillo King	La Vega
Hospital Presidente Estrella Ureña	Santiago
Hospital Immaculate Conception	Sánchez Ramírez
Hospital José Francisco Peña Gómez	Valverde
Hospital Matías Ramón Mella	Dajabón
Hospital Dr. Ricardo Limardo	Puerto Plata
Hospital Antonio Musa	San Pedro de Macorís
Hospital Provincial Dr. Ángel Contreras	Monte Plata

Participaram do estudo um total de 17 hospitais representando diferentes regiões geográficas da República Dominicana.

**Tabela 2:** – Características demográficas e clínicas basais da população do estudo (n = 469)

Variável	Valor
Idade materna, anos	26,71 ± 6,8
Obesidade pré-gestacional	112 (23,9)
Hipertensão crônica	81 (17,3)
Histórico familiar de pré-eclâmpsia	56 (12,0)
Histórico prévio de pré-eclâmpsia	42 (9,0)
Diabetes melito gestacional	18 (3,8)
Tabagismo atual	14 (3,0)

Os dados são apresentados como n (%) ou média ± desvio padrão, conforme apropriado.

### Fatores associados ao desenvolvimento de pré-eclâmpsia

Na análise bivariada, hipertensão crônica, pré-eclâmpsia prévia, histórico familiar de pré-eclâmpsia e obesidade pré-gestacional estiveram todos significativamente associados ao desenvolvimento de pré-eclâmpsia (todos p < 0,001). Mulheres com esses fatores de risco apresentaram chances significativamente maiores de desenvolver pré-eclâmpsia durante a gestação atual em comparação àquelas sem essas condições (Tabela 3). Os resultados da análise de regressão logística multivariável, incluindo AORs, IC95% e valores de p para todas as variáveis incluídas no modelo, são apresentados na Tabela 4.

**Tabela 3:** – Fatores de risco associados ao desenvolvimento de pré-eclâmpsia

Fator de risco	Pré-eclâmpsia, %	Sem pré-eclâmpsia, %	OR (IC95%)	Valor de p
Hipertensão crônica	44,15	16,87	3,91 (2,51-6,09)	< 0,001
Pré-eclâmpsia prévia	22,00	5,40	3,53 (2,24-5,56)	< 0,001
Histórico familiar de pré-eclâmpsia	24,00	8,70	3,01 (1,96-4,62)	< 0,001
Obesidade pré-gestacional	37,00	20,30	2,30 (1,43-3,70)	< 0,001

IC95%: intervalo de confiança de 95%; OR: odds ratio.

**Tabela 4:** – Análise de regressão logística multivariável dos fatores associados ao desenvolvimento de pré-eclâmpsia

Variável	AOR	IC95%	Valor de p
Idade materna (por incremento de 1 ano)	1,00	0,96-1,04	0,992
Histórico familiar de pré-eclâmpsia	3,75	1,64-8,55	0,002
Hipertensão crônica	1,33	0,66-2,67	0,420
Diabetes melito	0,46	0,09-2,35	0,349
Pré-eclâmpsia prévia	2,80	1,22-6,45	0,015
Obesidade pré-gestacional (IMC ≥ 30 kg/m^2^)	1,37	0,82-2,28	0,232

AOR: odds ratio ajustada; IC95%: intervalo de confiança de 95%; IMC: índice de massa corporal

### Impacto na morbidade e mortalidade materna e neonatal

Um total de quatro óbitos maternos hospitalares ocorreu durante o período do estudo, correspondendo a uma razão estimada de mortalidade materna hospitalar de 85,4 óbitos por 100.000 nascidos vivos. A taxa global de mortalidade fetal foi de 34 óbitos por 1.000 nascidos vivos.

Tanto a pré-eclâmpsia quanto a hipertensão crônica estiveram significativamente associadas a um risco aumentado de desfechos perinatais adversos, particularmente parto prematuro e baixo peso ao nascer. As associações detalhadas entre os DHGs e os desfechos maternos e neonatais são apresentadas na Tabela 5.

**Tabela 5:** – Associação da pré-eclâmpsia e da hipertensão crônica com desfechos maternos e neonatais adversos

Exposição/desfecho	Grupo exposto, %	Grupo controle, %	OR bruto (IC95%)	Valor de p
**Pré-eclâmpsia**				
Parto prematuro (< 37 semanas)	54,60	33,40	2,40 (1,62-3,55)	< 0,001
Baixo peso ao nascer	37,60	18,50	2,65 (1,73-4,07)	< 0,001
Descolamento prematuro de placenta	5,00	2,44	2,11 (0,68-6,54)	0,198
Mortalidade fetal/neonatal	4,00	1,63	2,52 (0,54-10,37)	0,174
**Hipertensão crônica**				
Parto prematuro (< 37 semanas)	65,35	32,00	3,99 (2,55-6,27)	< 0,001
Baixo peso ao nascer	33,33	20,00	1,99 (1,23-3,25)	0,030

IC95%: intervalo de confiança de 95%; OR: odds ratio.

## Discussão

Os achados deste estudo multicêntrico demonstram que os DHGs impõem uma carga epidemiológica e assistencial substancial à República Dominicana. A prevalência observada de pré-eclâmpsia (21,4%) foi superior às estimativas comumente relatadas na literatura internacional, que geralmente variam de 3% a 8% em países de alta renda. Essa diferença pode refletir uma maior carga de fatores de risco metabólicos e vasculares entre as mulheres atendidas no sistema público de saúde da República Dominicana, juntamente com barreiras estruturais ao acesso oportuno ao pré-natal, que limitam a implementação de estratégias preventivas baseadas em evidências, incluindo o início precoce de aspirina em baixa dose em gestantes de alto risco.^1,7,13^

Em consonância com a fisiopatologia estabelecida dos DHGs, nossa análise identificou hipertensão crônica, pré-eclâmpsia prévia, histórico familiar de pré-eclâmpsia e obesidade pré-gestacional como preditores significativos de pré-eclâmpsia. Entretanto, após o ajuste para potenciais fatores de confusão, apenas hipertensão crônica, histórico familiar de pré-eclâmpsia e obesidade pré-gestacional permaneceram independentemente associados ao desenvolvimento de pré-eclâmpsia, enquanto a pré-eclâmpsia prévia deixou de apresentar significância estatística. Esses achados reforçam a natureza multifatorial da pré-eclâmpsia e sustentam a importância tanto da suscetibilidade hereditária quanto da doença cardiometabólica preexistente em sua patogênese. A agregação familiar é considerada um reflexo de influências genéticas e epigenéticas que afetam a adaptação imunológica materna à gestação, o desenvolvimento placentário e as vias de sinalização angiogênica.^3,14,16^ Da mesma forma, hipertensão crônica e obesidade provavelmente contribuem por meio de disfunção endotelial, inflamação crônica, estresse oxidativo e remodelamento vascular prejudicado, criando, assim, um ambiente fisiológico que predispõe as mulheres ao desenvolvimento de pré-eclâmpsia.

Em relação à obesidade pré-gestacional, a análise univariável demonstrou associação significativa com a pré-eclâmpsia (OR, 2,30; IC95%, 1,43-3,70; p < 0,001), em consonância com a ampla literatura que identifica a obesidade como um importante fator contribuinte para inflamação sistêmica crônica, resistência à insulina e disfunção endotelial.^3,12^ Em nossa coorte, 42,5% das mulheres com obesidade pré-gestacional desenvolveram pré-eclâmpsia, em comparação com 34,7% daquelas sem obesidade. Entretanto, após o ajuste para potenciais fatores de confusão no modelo de regressão logística multivariável (Tabela 4), a obesidade pré-gestacional deixou de estar independentemente associada à pré-eclâmpsia (AOR, 1,37; IC95%, 0,82-2,28; p = 0,231). Essa atenuação sugere que o efeito da obesidade pode ser mediado, pelo menos em parte, por sua estreita relação com outros fatores de risco cardiometabólicos, particularmente a hipertensão crônica, em vez de representar um determinante totalmente independente da pré-eclâmpsia nesta coorte.

Os desfechos neonatais adversos observados, particularmente parto prematuro e baixo peso ao nascer, refletem tanto a gravidade da doença quanto a frequente necessidade de antecipação médica do parto para prevenir complicações maternas e fetais.^5,13^ Mais da metade das mulheres com pré-eclâmpsia apresentou parto prematuro, enfatizando a substancial carga obstétrica associada a essa condição. Na prática clínica, o parto prematuro frequentemente representa a única intervenção definitiva capaz de prevenir a progressão para complicações potencialmente fatais, como eclâmpsia, descolamento prematuro de placenta, síndrome HELLP e morte materna.^14^ Além disso, a elevada prevalência de baixo peso ao nascer é consistente com a insuficiência placentária decorrente de isquemia uteroplacentária crônica, que compromete o fornecimento de oxigênio e nutrientes ao feto e contribui para a restrição do crescimento fetal.^14,16^

Um achado importante deste estudo foi a razão estimada de mortalidade materna hospitalar de 85,4 óbitos por 100.000 nascidos vivos, que permanece substancialmente superior às metas estabelecidas pelos Objetivos de Desenvolvimento Sustentável. De forma semelhante, a taxa de mortalidade fetal de 34 óbitos por 1.000 nascidos vivos ressalta a persistente carga de desfechos perinatais adversos associados aos DHGs. Em conjunto, esses achados sugerem deficiências persistentes no diagnóstico precoce, na vigilância pré-natal, no encaminhamento oportuno e no acesso à assistência obstétrica especializada.^2,4,8^

Sob a perspectiva da saúde pública, nossos achados reforçam a necessidade de fortalecer os serviços de pré-natal, aprimorar a identificação precoce de mulheres de alto risco e garantir acesso oportuno ao manejo obstétrico multidisciplinar.^6,9^ Historicamente, os DHGs eram considerados condições restritas à gestação, que se resolviam após o parto. No entanto, evidências acumuladas indicam que a gestação funciona como um teste natural de estresse cardiovascular, e que a pré-eclâmpsia representa uma manifestação precoce de uma predisposição subjacente à doença cardiovascular. Mulheres com histórico de pré-eclâmpsia apresentam risco significativamente aumentado, em longo prazo, de hipertensão crônica, doença cardíaca isquêmica, insuficiência cardíaca e acidente vascular cerebral. Assim, o acompanhamento coordenado no período pós-parto, envolvendo tanto obstetras quanto cardiologistas, representa uma oportunidade fundamental para identificar mulheres com maior risco cardiovascular e implementar estratégias preventivas que possam melhorar os desfechos maternos em longo prazo.^10-12,17,18^

### Limitações do estudo

Algumas limitações deste estudo devem ser reconhecidas. Em primeiro lugar, seu delineamento transversal impede o estabelecimento de relações temporais ou causais entre os fatores de risco identificados e o desenvolvimento da pré-eclâmpsia. Em segundo lugar, a coleta de parte dos dados referentes ao histórico clínico e obstétrico por meio de entrevistas estruturadas pode ter introduzido viés de recordação ou de relato. Entretanto, essas limitações são parcialmente compensadas pelo delineamento multicêntrico do estudo, que incluiu 17 hospitais distribuídos por toda a República Dominicana. Até onde sabemos, esta representa uma das maiores investigações epidemiológicas sobre DHGs já conduzidas no país e uma das mais representativas em termos de cobertura geográfica.

## Conclusões

Os DHGs permanecem como uma importante causa de morbidade e mortalidade materna e neonatal no sistema público de saúde da República Dominicana.

Na análise multivariável, hipertensão crônica, histórico familiar de pré-eclâmpsia e obesidade pré-gestacional foram identificados como preditores independentes de pré-eclâmpsia.

A pré-eclâmpsia e a hipertensão crônica estiveram associadas a um risco aumentado de desfechos perinatais adversos, particularmente parto prematuro e baixo peso ao nascer, ressaltando a importância da identificação precoce do risco, da vigilância pré-natal direcionada e do manejo multidisciplinar oportuno para melhorar os desfechos maternos e neonatais.
