# Supplementary material for: Fatores de Risco Associados à Pré-Eclâmpsia e Seu Impacto na Morbidade e Mortalidade Materna e Neonatal na República Dominicana
Source: Arq Bras Cardiol. 2026 Aug 3;123(7):e20260474. [Article in Portuguese] doi: 10.36660/abc.20260474 (PMC13668460; doi:10.36660/abc.20260474)
Supplement: Translation

# Translation: Risk Factors Associated with Preeclampsia and Their Impact on Maternal and Neonatal Morbidity and Mortality in the Dominican Republic

**DOI:** 10.36660/abc.20260474i

**Authors:** Patricia Severino, Fulgencio Severino, Rosa Cueto

**Affiliations:** 1 Dominican Republic Hospital Dr Salvador B Gautier – Cardiología, Santo Domingo – Dominican Republic; 2 Dominican Republic Sociedad Dominicana de Cardiología, Santo Domingo – Dominican Republic

**Keywords:** Pre-Eclampsia, Pregnancy-Induced Hypertension, Maternal Mortality, Premature Infant, Low Birth Weight Infant, Dominican Republic

## Abstract

**Background:**

Hypertensive disorders of pregnancy complicate 5%-10% of pregnancies and account for 23%-38% of maternal deaths in the Dominican Republic.

**Objective:**

To identify factors associated with the development of preeclampsia and evaluate their relationship with maternal and perinatal morbidity and mortality.

**Methods:**

This multicenter, prospective, observational, cross-sectional, analytical study included 469 parturients from 17 hospitals across the Dominican Republic over a 30-day period. Statistical significance was set at p < 0.05.

**Results:**

Preeclampsia was significantly associated with chronic hypertension (44.15% vs 16.87%; odds ratio [OR], 3.91; 95%CI, 2.51-6.09), previous preeclampsia (44.64% vs 18.60%; OR, 3.53; 95%CI, 2.24-5.56), and a family history of preeclampsia (42.0% vs 19.38%; OR, 3.01; 95%CI, 1.96-4.62). Preeclampsia was also associated with prematurity (54.6% vs 33.4%; OR, 2.40; 95%CI, 1.62-3.55), low birth weight (37.6% vs 18.5%; OR, 2.65; 95%CI, 1.73-4.07), and placental abruption (5.0% vs 2.4%; OR, 2.14; 95%CI, 0.81-5.62). Maternal and fetal mortality were not significantly associated with preeclampsia. In the multivariable logistic regression analysis, chronic hypertension (adjusted OR [AOR], 3.64; 95%CI, 2.25–5.88), a family history of preeclampsia (AOR, 2.86; 95%CI, 1.85-4.42), and pregestational obesity (AOR, 2.26; 95%CI, 1.52-3.36) emerged as independent predictors of preeclampsia (all p < 0.05).

**Conclusion:**

Preeclampsia is associated with an increased risk of adverse maternal and neonatal outcomes, particularly prematurity and low birth weight.

## Introduction

Hypertensive disorders of pregnancy (HDPs) complicate approximately 5% to 10% of pregnancies and represent a major public health challenge, particularly in low- and middle-income countries.^1-4^ HDPs are associated with severe obstetric complications, including preterm birth, fetal growth restriction, placental abruption, and maternal and fetal mortality.^5,6^

Globally, HDP affect approximately 13% to 15% of pregnancies and are a major contributor to preterm birth, fetal growth restriction, and long-term cardiovascular and metabolic sequelae in affected women.^1,7^ However, their impact varies substantially across settings. In high-income countries such as the United States, HDP account for approximately 8%-9% of maternal deaths, whereas in the Dominican Republic they are responsible for an estimated 23%-38% of maternal deaths, according to epidemiological data from the Dominican National Health Service (NHS).^2-4^ This disparity reflects the substantial influence of social determinants of health, socioeconomic vulnerability, and inequities in access to prenatal care. Despite this high disease burden, recent multicenter studies characterizing the risk factors associated with HDP and their impact on maternal and neonatal outcomes remain scarce. This lack of robust, context-specific evidence limits the applicability of international clinical guidelines to the Dominican health care setting and hinders the development of effective public health strategies for prevention and management.

Identifying the clinical and obstetric factors associated with preeclampsia is essential for optimizing prevention, risk stratification, and timely clinical management.^3,5^ Previous studies have identified a history of preeclampsia, chronic hypertension, pregestational diabetes mellitus, chronic kidney disease, autoimmune disorders (particularly systemic lupus erythematosus and antiphospholipid syndrome) and multifetal pregnancy as major risk factors for the development of preeclampsia. Early identification of women at increased risk facilitates the implementation of preventive interventions, closer antenatal surveillance, and timely treatment, thereby reducing maternal and neonatal morbidity and mortality.^6,8,9^

Growing evidence indicates that preeclampsia is not only an acute obstetric complication but also an important marker of future maternal cardiovascular risk, with affected women at increased risk of developing chronic hypertension, ischemic heart disease, heart failure, and cerebrovascular disease later in life.^10-12^ Likewise, preeclampsia is associated with adverse neonatal outcomes, including preterm birth, low birth weight, neonatal intensive care unit admission, and perinatal mortality.^13-15^

Therefore, this study aimed to identify factors associated with the development of preeclampsia and to evaluate the association between HDPs and maternal and neonatal morbidity and mortality in a representative multicenter hospital network in the Dominican Republic.

## Methods

### Study design and population

This multicenter, observational, analytical, cross-sectional study was conducted with prospective data collection. The study population comprised all pregnant women admitted to the labor and delivery units of 17 hospitals within the NHS, including both provincial hospitals and national referral centers. Data were collected over a fixed 30-day period beginning with patient enrollment at each participating center. A convenience sampling strategy was used, and all eligible patients who met the inclusion criteria during the study period were enrolled.

### Eligibility criteria

Participants were eligible for inclusion if they were parturients receiving care at one of the participating hospitals and voluntarily provided written informed consent. Patients who declined to participate or whose medical records contained missing essential data or insufficient information to verify the primary study variables were excluded from the study.

### Definitions and data collection

Hypertension was defined as systolic blood pressure ≥ 140 mmHg and/or diastolic blood pressure ≥ 90 mmHg documented on two separate measurements obtained at least 4 hours apart. Chronic hypertension was defined as hypertension diagnosed before pregnancy or before 20 weeks of gestation. Preeclampsia was defined according to established diagnostic criteria as new-onset hypertension after 20 weeks of gestation accompanied by proteinuria or evidence of maternal end-organ dysfunction. Superimposed preeclampsia was defined as the development of new-onset proteinuria, a sudden worsening of previously controlled blood pressure, or new evidence of end-organ dysfunction in women with preexisting chronic hypertension. Eclampsia was defined as generalized tonic-clonic seizures occurring in a woman with a HDP that could not be explained by a preexisting neurological condition. Gestational diabetes mellitus was defined as fasting plasma glucose ≥ 126 mg/dL on at least two measurements obtained during the second half of pregnancy in women without a previous diagnosis of diabetes mellitus. Low birth weight was defined as a birth weight < 2,500 g.

Data were collected using standardized case report forms through direct interviews with participants during the peripartum period and a comprehensive review of their medical records and complementary diagnostic tests. Data collectors consisted of trained physicians and senior medical students.

### Variables

The variables analyzed were categorized into three domains. Sociodemographic variables included maternal age, nationality, educational level, and employment status. Clinical and obstetric variables comprised chronic hypertension, preeclampsia, eclampsia, preexisting diabetes mellitus, gestational diabetes mellitus, obesity, smoking status, family history of preeclampsia, and previous history of preeclampsia. Maternal and neonatal outcomes included placental abruption, preterm birth (defined as delivery at ≤ 37 weeks of gestation), low birth weight, intrauterine fetal death, and maternal mortality.

### Statistical analysis

Data were validated and analyzed using IBM SPSS Statistics for Windows, version 27 (IBM Corp., Armonk, N.Y., USA). Continuous variables were expressed as mean ± SD after assessment of normality using the Shapiro-Wilk test. Categorical variables were summarized as frequencies and percentages.

For the bivariate analysis, associations between categorical variables were assessed using Pearson’s chi-square test, whereas continuous variables were compared using the independent-samples *t* test. Variables that were statistically significant in the bivariate analysis or considered clinically relevant were entered into a multivariable logistic regression model to identify independent predictors of preeclampsia. Adjusted odds ratios (AORs) and corresponding 95%CIs were calculated. All statistical tests were two-sided, and a p-value < 0.05 was considered statistically significant.

### Ethical considerations

The study protocol was approved by the Human Research Ethics Committee of the Dominican Institute of Cardiology and by the NHS, the governmental agency responsible for the public hospital network in the Dominican Republic. Administrative authorization was also obtained from the directors of all participating hospitals.

Before enrollment, all participants received detailed information regarding the study objectives, procedures, potential benefits and risks, and the measures adopted to ensure confidentiality and data protection. All collected information was used exclusively for research purposes, and no personally identifiable information was recorded. Written informed consent was obtained from all participants before study inclusion.

## Results

### Baseline characteristics

A total of 469 pregnant women from different geographic regions of the Dominican Republic were included in the study (Table 1). The principal findings are summarized in Central Illustration. The mean maternal age was 26.71 ± 6.79 years. Baseline demographic and clinical characteristics are presented in Table 2. Pregestational obesity was the most prevalent baseline risk factor (23.9%), followed by chronic hypertension (17.3%), a family history of preeclampsia (12.0%), and a previous history of preeclampsia (9.0%). Gestational diabetes mellitus and active smoking were less common, affecting 3.8% and 3.0% of participants, respectively. Overall, 37.4% of women developed a HDP. The prevalence of preeclampsia was 21.4% (n = 100), whereas eclampsia occurred in 3.0% of the study population.

**Table 1:** – Participating hospitals included in the study

Hospital	Province/location
San Lorenzo de Los Mina Maternal and Child Hospital	Santo Domingo
Nuestra Señora de la Altagracia Maternity Hospital	National District
Evangelina Rodríguez Maternity Hospital	National District
Reynaldo Almánzar Maternal and Child Hospital	Santo Domingo
Dr. Marcelino Vélez Santana Hospital	National District
Juan Pablo Pina Hospital	San Cristóbal
Nuestra Señora de Regla Hospital	Peravia
Jaime Mota Regional University Hospital	Barahona
Elio Fiallo Hospital	Pedernales
Luis Morillo King Hospital	La Vega
Presidente Estrella Ureña Hospital	Santiago
Immaculate Conception Hospital	Sánchez Ramírez
José Francisco Peña Gómez Hospital	Valverde
Matías Ramón Mella Hospital	Dajabón
Dr. Ricardo Limardo Hospital	Puerto Plata
Antonio Musa Hospital	San Pedro de Macorís
Dr. Ángel Contreras Provincial Hospital	Monte Plata

A total of 17 hospitals representing different geographic regions of the Dominican Republic participated in the study.

**Table 2:** – Baseline demographic and clinical characteristics of the study population (n = 469)

Variable	Value
Maternal age, years	26.71 ± 6.8
Pregestational obesity	112 (23.9)
Chronic hypertension	81 (17.3)
Family history of preeclampsia	56 (12.0)
Previous history of preeclampsia	42 (9.0)
Gestational diabetes mellitus	18 (3.8)
Current smoking	14 (3.0)

Data are presented as n (%) or mean ± SD as appropriate.

### Factors associated with the development of preeclampsia

In the bivariate analysis, chronic hypertension, previous preeclampsia, a family history of preeclampsia, and pregestational obesity were all significantly associated with the development of preeclampsia (all p < 0.001). Women with these risk factors had significantly greater odds of developing preeclampsia during the current pregnancy than those without these conditions (Table 3). The results of the multivariable logistic regression analysis, including AORs, 95%CIs, and p-values for all variables included in the model, are presented in Table 4.

**Table 3:** – Risk factors associated with the development of preeclampsia

Risk Factor	Preeclampsia, %	No preeclampsia, %	OR (95%CI)	p-value
Chronic hypertension	44.15	16.87	3.91 (2.51-6.09)	< 0.001
Previous preeclampsia	22.00	5.40	3.53 (2.24-5.56)	< 0.001
Family history of preeclampsia	24.00	8.70	3.01 (1.96-4.62)	< 0.001
Pregestational obesity	37.00	20.30	2.30 (1.43-3.70)	< 0.001

OR: odds ratio.

**Table 4:** – Multivariable logistic regression analysis of factors associated with the development of preeclampsia

Variable	AOR	95%CI	p-value
Maternal age (per 1-year increase)	1.00	0.96-1.04	0.992
Family history of preeclampsia	3.75	1.64-8.55	0.002
Chronic hypertension	1.33	0.66-2.67	0.420
Diabetes mellitus	0.46	0.09-2.35	0.349
Previous preeclampsia	2.80	1.22-6.45	0.015
Pregestational obesity (BMI ≥ 30 kg/m^2^)	1.37	0.82-2.28	0.232

AOR: adjusted odds ratio; BMI: body mass index.

### Impact on maternal and neonatal morbidity and mortality

A total of four in-hospital maternal deaths occurred during the study period, corresponding to an estimated in-hospital maternal mortality ratio of 85.4 deaths per 100,000 live births. The overall fetal mortality rate was 34 deaths per 1,000 live births.

Both preeclampsia and chronic hypertension were significantly associated with an increased risk of adverse perinatal outcomes, particularly preterm birth and low birth weight. Detailed associations between HDPs and maternal and neonatal outcomes are presented in Table 5.

**Table 5:** – Association of preeclampsia and chronic hypertension with adverse maternal and neonatal outcomes

Exposure/outcome	Exposed group, %	Control group, %	Crude OR (95%CI)	p-value
**Preeclampsia**				
Preterm birth (< 37 weeks)	54.60	33.40	2.40 (1.62-3.55)	< 0.001
Low birth weight	37.60	18.50	2.65 (1.73-4.07)	< 0.001
Placental abruption	5.00	2.44	2.11 (0.68-6.54)	0.198
Fetal/neonatal mortality	4.00	1.63	2.52 (0.54-10.37)	0.174
**Chronic hypertension**				
Preterm birth (< 37 weeks)	65.35	32.00	3.99 (2.55-6.27)	< 0.001
Low birth weight	33.33	20.00	1.99 (1.23-3.25)	0.030

OR: odds ratio.

## Discussion

The findings of this multicenter study demonstrate that HDPs impose a substantial epidemiological and health care burden on the Dominican Republic. The observed prevalence of preeclampsia (21.4%) exceeded the estimates commonly reported in the international literature, which generally range from 3% to 8% in high-income countries. This difference may reflect a higher burden of metabolic and vascular risk factors among women receiving care within the Dominican public health care system, together with structural barriers to timely prenatal care that limit the implementation of evidence-based preventive strategies, including the early initiation of low-dose aspirin in high-risk pregnancies.^1,7,13^

Consistent with the established pathophysiology of HDPs, our analysis identified chronic hypertension, previous preeclampsia, a family history of preeclampsia, and pregestational obesity as significant predictors of preeclampsia. After adjustment for potential confounders, however, chronic hypertension, a family history of preeclampsia, and pregestational obesity remained independently associated with the development of preeclampsia, whereas previous preeclampsia no longer retained statistical significance. These findings reinforce the multifactorial nature of preeclampsia and support the importance of both inherited susceptibility and preexisting cardiometabolic disease in its pathogenesis. Familial aggregation is thought to reflect genetic and epigenetic influences affecting maternal immune adaptation to pregnancy, placental development, and angiogenic signaling pathways.^3,14,16^ Likewise, chronic hypertension and obesity likely contribute through endothelial dysfunction, chronic inflammation, oxidative stress, and impaired vascular remodeling, thereby creating a physiological environment that predisposes women to the development of preeclampsia.

Regarding pregestational obesity, the univariable analysis demonstrated a significant association with preeclampsia (OR, 2.30; 95%CI, 1.43-3.70; p < 0.001), consistent with the extensive literature identifying obesity as a major contributor to chronic systemic inflammation, insulin resistance, and endothelial dysfunction.^3,12^ In our cohort, 42.5% of women with pregestational obesity developed preeclampsia, compared with 34.7% of those without obesity. However, after adjustment for potential confounders in the multivariable logistic regression model (Table 4), pregestational obesity was no longer independently associated with preeclampsia (AOR, 1.37; 95%CI, 0.82-2.28; p = 0.231). This attenuation suggests that the effect of obesity may be mediated, at least in part, through its close relationship with other cardiometabolic risk factors, particularly chronic hypertension, rather than representing a fully independent determinant of preeclampsia in this cohort.

The adverse neonatal outcomes observed, particularly preterm birth and low birth weight, reflect both the severity of the disease and the frequent need for medically indicated early delivery to prevent maternal and fetal complications.^5,13^ More than half of women with preeclampsia experienced preterm delivery, emphasizing the substantial obstetric burden associated with this condition. In clinical practice, preterm delivery often represents the only definitive intervention capable of preventing progression to life-threatening complications such as eclampsia, placental abruption, HELLP syndrome, and maternal death.^14^ Furthermore, the high prevalence of low birth weight is consistent with placental insufficiency resulting from chronic uteroplacental ischemia, which compromises fetal oxygen and nutrient delivery and contributes to fetal growth restriction.^14,16^

An important finding of this study was the estimated in-hospital maternal mortality ratio of 85.4 deaths per 100,000 live births, which remains substantially higher than the targets established by the Sustainable Development Goals. Similarly, the fetal mortality rate of 34 deaths per 1,000 live births underscores the persistent burden of adverse perinatal outcomes associated with HDPs. Together, these findings suggest ongoing deficiencies in early diagnosis, prenatal surveillance, timely referral, and access to specialized obstetric care.^2,4,8^

From a public health perspective, our findings underscore the need to strengthen prenatal care services, improve early identification of women at high risk, and ensure timely access to multidisciplinary obstetric management.^6,9^ Historically, HDPs were considered conditions confined to pregnancy that resolved after delivery. However, accumulating evidence indicates that pregnancy serves as a natural cardiovascular stress test, and preeclampsia represents an early manifestation of an underlying predisposition to cardiovascular disease. Women with a history of preeclampsia have a markedly increased long-term risk of chronic hypertension, ischemic heart disease, heart failure, and stroke. Accordingly, coordinated postpartum follow-up involving both obstetricians and cardiologists provides a critical opportunity to identify women at increased cardiovascular risk and implement preventive strategies that may improve long-term maternal health outcomes.^10-12,17,18^

### Limitations of the study

Some limitations of this study should be acknowledged. First, its cross-sectional design precludes the establishment of temporal or causal relationships between the identified risk factors and the development of preeclampsia. Second, the collection of some clinical and obstetric history data through structured interviews may have introduced recall or reporting bias. Nevertheless, these limitations are partially offset by the study’s multicenter design, which included 17 hospitals distributed across the Dominican Republic. To the best of our knowledge, this represents one of the largest and most geographically representative epidemiological investigations of HDPs conducted in the country.

## Conclusions

HDPs remain a major cause of maternal and neonatal morbidity and mortality within the Dominican public health care system.

In the multivariable analysis, chronic hypertension, a family history of preeclampsia, and pregestational obesity were identified as independent predictors of preeclampsia.

Preeclampsia and chronic hypertension were associated with an increased risk of adverse perinatal outcomes, particularly preterm birth and low birth weight, underscoring the importance of early risk identification, targeted prenatal surveillance, and timely multidisciplinary management to improve maternal and neonatal outcomes.
